# Reductions in kinesin expression are associated with nitric oxide-induced axonal damage

**DOI:** 10.1002/jnr.23556

**Published:** 2015-01-29

**Authors:** Juliana Redondo, Kelly Hares, Alastair Wilkins, Neil Scolding, Kevin Kemp

**Affiliations:** 1 Multiple Sclerosis and Stem Cell Group, School of Clinical Sciences, University of BristolBristol, United Kingdom

**Keywords:** axonal transport, nitrosative stress, motor proteins, mesenchymal stem cells, neuroprotection

## Abstract

Axonal injury is often characterized by axonal transport defects and abnormal accumulation of intra-axonal components. Nitric oxide (NO) has a key role in mediating inflammatory axonopathy in many neurodegenerative diseases, but little is known about how nitrosative/oxidative stress affects axonal transport or whether reductions in kinesin superfamily protein (KIF) expression correlate with axon pathology. KIFs are molecular motors that have a key role in axonal and dendritic transport, and impairment of these mechanisms has been associated with a number of neurological disorders. This study shows that rat cortical neurons exposed to NO display both a time-dependent decrease in KIF gene/protein expression and neurofilament phosphorylation in addition to a reduction in axonal length and neuronal survival. Because mesenchymal stem cells (MSCs) represent a promising therapeutic candidate for neuronal/axonal repair, this study analyzes the capacity of MSCs to protect neurons and axonal transport mechanisms from NO damage. Results show that coculture of MSCs with NO-exposed neurons results in the preservation of KIF expression, axonal length, and neuronal survival. Altogether, these results suggest a potential mechanism involved in the disruption of axonal transport and abnormal accumulation of proteins in axons during nitrosative insult. We hypothesize that impaired axonal transport contributes, per se, to progression of injury and provide further evidence of the therapeutic potential of MSCs for neurodegenerative disorders. © 2015 The Authors. Journal of Neuroscience Research Published by Wiley Periodicals, Inc.

Axonal damage is a core pathological feature of many neurodegenerative disorders (Medana and Esiri, 2003). Regardless of the underlying cause, axonal injury is often characterized by a combination of axonal transport defects, axonal swellings, neurofilament (NF) dephosphorylation, oxidative/nitrosative stress, and mitochondrial dysfunction (Medana and Esiri, 2003; Coleman, 2005).

Abnormal accumulation of intra-axonal components, such as amyloid precursor protein (APP) and NF, are hallmarks of several neurodegenerative disorders, and some authors hypothesize that such accumulations are associated with disruption of axonal transport (Stokin et al., 2005; Kreutzer et al., 2012). Intact axonal transport is required to maintain the integrity of axons and the overall function of the neuron. The kinesin superfamily proteins (KIFs) are a large family of molecular motors that, by using microtubules as rails and ATP to generate motile force, selectively transport molecules, such as membranous organelles, protein complexes, and mRNA, throughout axons and dendrites.

Mounting evidence has highlighted a role for the dysregulation of KIFs in neurological disorders. KIF5A and KIF21B have been particularly implicated. The KIF5A subtype is a neuron-specific kinesin that transports cargos, such as NF, APP, synaptic vesicle precursors, and mitochondria (Hirokawa et al., 2009). Mutations in the KIF5A gene have been linked to hereditary spastic paraplegia (Reid et al., 2002; Musumeci et al., 2011), and its downregulation has been associated with axonal transport defects in models of multiple sclerosis (MS; Kreutzer et al., 2012), Alzheimer's disease (Stokin et al., 2005), and Parkinson's disease (Chu et al., 2012).

KIF21B expression is detected in brain, eye, and spleen. In neurons, KIF21B is enriched in dendrites; its function is still unknown but could be to deliver cargos to the distal regions of dendrites, where the microtubule polarity is mixed (Marszalek et al., 1999). Recently, a genome-wide association screen for MS correlated a single-nucleotide polymorphism located in the KIF21B intron with the disease, establishing this kinesin as a susceptibility locus for MS (Goris et al., 2010). Furthermore, a recent study has shown reductions in KIF5A and KIF21B mRNA in gray matter of MS patients, which may have relevance to the axonal pathology seen in the disease (Hares et al., 2013).

Nitric oxide (NO) has a key role in mediating inflammatory axonopathy in MS through the promotion of protein misfolding, disruption of mitochondrial respiratory chain, organelle fragmentation, lipid peroxidation, and matrix metalloproteinases activation, leading to axon damage (Smith and Lassmann, 2002; Knott and Bossy-Wetzel, 2009). This study investigates the effect of NO exposure on KIF gene and protein expression and NF phosphorylation, crucial for axon stability. The current study also evaluates whether KIF expression correlates with axon damage and pathology.

There is increasing interest in cell-based therapies as new therapeutic approaches for several neurological diseases. Specifically, human bone marrow-derived mesenchymal stem cells (MSCs) represent a promising candidate for neuronal protection and are currently being tested in clinical trials. These cells possess paracrine activities that may rescue neurons from apoptosis (Chen et al., 2003), have anti-inflammatory properties (Lanza et al., 2009), and secrete neurotrophic (Wilkins et al., 2009) and antioxidant (Kemp et al., 2010) factors. The current study, therefore, analyzes the capacity of MSCs to protect neurons and axonal transport mechanisms in rodent cortical neurons exposed to NO.

## MATERIALS AND METHODS

### Neuronal Cell Culture

Neuronal cultures were prepared from cortices of embryonic day 18 rat embryos as previously described (Wilkins et al., 2001). This study received institutional approval (University of Bristol; University Investigation No. UB/07/016), and experiments were performed in accordance with the Animals (Scientific Procedures) Act 1986 of the United Kingdom. In brief, the pregnant female was sacrificed, and embryos were removed. Cortices were isolated, and meninges were removed. Enzymatic and mechanical dissociation were used to obtain a clear cell suspension. Cells were then counted and were plated onto poly-L-lysine-coated 13-mm coverslips at 2.5 × 10^5^/coverslip in 24-well plates or in poly-L-lysine-coated six-well plates at 2 × 10^6^/well. Cortical cells were cultured in Dulbecco's modified Eagles medium (DMEM; Sigma-Aldrich, St. Louis, MO) supplemented with 2% B27 (Gibco, Paisley, United Kingdom) and 1% penicillin and streptomycin (Sigma-Aldrich). Five days after plating, neurons identified by βIII-tubulin staining represented 97.9% ± 1.2% of the total cells (n = 3). The remaining cells were predominantly glial fibrillary acidic protein-expressing astrocytes and GalC-expressing oligodendrocytes. At 5 days of in vitro culture, cortical neurons were exposed to experimental conditions. The base medium for all experiments was serum-free minimal medium (MIN), which consisted of DMEM supplemented with 1% insulin-free Sato (containing 100 μg/ml bovine serum albumin [BSA], 100 μg/ml transferrin, 0.06 μg/ml progesterone, 16 μg/ml putrescine, 0.04 μg/ml selenite, 0.04 μg/ml thyroxine, and 0.04 μg/ml triiodothryonine), 1% penicillin and streptomycin, and 0.5% L-glutamine.

### NO Insult to Neurons

A stock solution (50 mM in 10 mM NaOH) of (Z)−1-[2-(aminoethyl)-*N*-(2-ammonioethyl)amino]daizen-1-ium-1,2-diolate (DETANONOate; Alexis Biochemicals, San Diego, CA) was prepared immediately before use. After 5 days in vitro culture, medium was removed from all wells, cells were washed in DMEM, and MIN or MIN with the addition of the NO donor DETANONOate 0.1 mM (MIN + NO) was added to appropriate wells for 1, 2, 4, 6, or 24 hr. The time course of cell death after NO exposure was calculated by counting cortical neurons costained with the neuronal marker βIII-tubulin and 4′,6-diamidino-2-phenylindole (DAPI) for nuclear identification. Cells with fragmented nuclei or with an abnormal shape and size were excluded from the counting. Nine random fields per culture treatment and at least three culture repeats per treatment were analyzed.

### Immunocytochemistry

Neuronal cultures were stained after fixation with 4% paraformalaldehyde and permeabilization with 100% ice-cold methanol at −20°C for 10 min and were blocked with phosphate-buffered saline (PBS)/5% normal goat serum/0.1% Triton for 30 min. Primary antibodies used were the panaxonal NF marker SMI312 (1:600; lot No. LN14971402; Sternberger-Cambridge Biosciences); the NF marker NF 70 kD that recognizes the low-molecular-weight subunit of the NF triplet, which is phosphate independent (1:400; lot No. 21110242; Chemicon; Temecula, CA); the neuronal marker βIII-tubulin (1:400; lot No. 063M4776; Promega, Madison, WI), KIF5A (1:250; lot No. R04153; Sigma-Aldrich), and KIF21B (1:250; lot No. R12701; Sigma-Aldrich). Species-specific Alexa Fluor 488- and 555-conjugated secondary antibodies (1:500; Invitrogen, Carlsbad, CA) were used to visualize primary antibody staining. DAPI Vectashield (H-1200; Vector Laboratories, Burlingame, CA) was used for nuclear identification.

### Axon Length and Cell Survival Assay

After 5 days of in vitro culture, cortical neurons were exposed to MIN, MIN with NO (MIN + NO), and MIN with NO and MSCs (MIN + NO + MSC) in a transwell coculture system. Evaluation of axon length and neuronal cell survival was carried out with immunocytochemistry for the panaxonal NF marker SMI312, NF 70 kD, the neuronal marker βIII-tubulin, and DAPI for nuclear identification. Average axon length per field was calculated by measuring the length of SMI312- and NF 70 kD-positive axons per field, and costaining of βIII-tubulin/DAPI was used to identify live nuclei for cell survival counts. Cells with fragmented nuclei or with an abnormal shape or size were excluded from the counting. ImageJ software was used to measure axon length and to count cell nuclei. Nine random fields per culture treatment and at least three culture repeats per treatment were analyzed.

### Western Blot and Dot Blot Analysis

Neurons were cultured at 2 × 10^6^/well in a six-well plate for 5 days before exposure to NO as previously described. At set time points, cells were washed and lysed with Beadlyte cell signaling universal lysis buffer (Upstate, Lake Placid, NY). All protein samples were quantified with a Qubit Fluorometer and a Quant-iT protein assay kit (Invitrogen) according to the manufacturer's instructions to ensure equal loading of samples. Western blot analysis was performed as previously described (Kemp et al., 2011). In brief, lysates were heated to 95°C for 5 min with Laemmli 2× sample buffer (Invitrogen, Paisley, United Kingdom) and run on Tris-HCl 10–20% ready gels (Bio-Rad, Hercules, CA). After having been transferred to nitrocellulose membrane (Bio-Rad, Hertfordshire, United Kingdom) and blocked in 5% BSA (Sigma-Aldrich)/Tris-buffered saline-Tween (TBS-T; Bio-Rad) for 1 hr, membranes were incubated overnight in primary antibody at 4°C (in 5% BSA/TBS-T). Antibodies used were rabbit anti-KIF5A (1:15,000; lot No. R04153; Sigma-Aldrich), rabbit anti-KIF21B (1:15,000; lot No. R12701; Sigma-Aldrich), mouse antiglyceraldehyde-3-phosphate dehydrogenase (GAPDH; 1:10,000; Abcam, Cambridge, MA), and rabbit antineuronal specific enolase (NSE; 1:5,000; lot No. GR61106-22; Abcam). Values are expressed relative to the loading control proteins GAPDH and NSE.

For dot blot experiments, lysates were diluted 1:200 in TBS, and 100 µl of sample was added to the Bio-Dot Microfiltration apparatus containing a prewet nitrocellulose membrane (Bio-Rad). The samples were then transferred to the membrane for 1 hr by gravity filtration. After having been transferred to nitrocellulose membrane and blocked in 5% BSA/TBS-T, membranes were incubated overnight in primary antibody at 4°C. Antibodies used were rabbit anti-KIF5A (1:15,000; lot No. R04153; Sigma-Aldrich), rabbit anti-KIF21B (1:15,000; lot No. R12701; Sigma-Aldrich), mouse anti-NF200 (1:10,000; Sigma-Aldrich), mouse anti-SMI312 (1:3,000; Sternberger-Cambridge Biosciences), mouse anti-GAPDH (1:10,000; Abcam), and rabbit anti-NSE (1:5,000; lot No. GR61106-22; Abcam).

Immunoreactivity was detected with secondary anti-rabbit (1:3,000) or anti-mouse (1:5,000) horseradish peroxidase-conjugated antibodies (Abcam) in 5% BSA/TBS-T, and specific protein expression patterns were visualized by chemiluminescence with an ECL Plus Western blotting detection system (Amersham Biosciences, Piscataway, NJ). After development, ImageJ (NIH) was used to measure the integrated density of Western blot bands and dot blot samples.

### Real-Time Polymerase Chain Reaction

RNA was extracted, and cDNA was produced as previously described (Kemp et al., 2011) with the Taqman Gene Expression Cells to Ct kit (Applied Biosystems, Foster City, CA) according to the manufacturer's instructions. At set time points, cells were washed in PBS, lysis solution plus DNAse was added, and cells were detached with a scraper. After 5 min, stop solution was added for 2 min, and samples were stored at −80°C until use. All RNA samples were quantified with a Qubit Fluorometer and a Quant-iT RNA assay kit (Invitrogen) according to the manufacturer's instructions to ensure equal loading of RNA samples. To synthesize cDNA, 100 ng extracted RNA was added to the reverse transcription buffer and RT enzyme mix, placed in a thermal cycler, and incubated at 37°C for 1 hr and 95°C for 5 min. RT-PCR was performed with the StepOnePlus real-time PCR System (Applied Biosystems) with assay-on-demand gene expression products for KIF5A, KIF21B, and 18S rRNA (Taqman MGB probe; FAM dye labeled; Applied Biosystems) with 10 ng cDNA in a total volume of 20 µl master mix. Reactions were run at 50°C for 2 min, 95°C for 10 min, and 40 cycles of 95°C for 15 sec and 60°C for 1 min. All samples were analyzed in triplicate. The relative gene expression (RQ value) of KIF5A and KIF21B was calculated with the 2^–ΔΔCt^ method, and the mean was taken for each group. 18S rRNA was used as the reference “housekeeping” gene.

### Establishment of Human MSC Cultures

Bone marrow samples were obtained by an orthopedic surgeon at Southmead Hospital, Bristol, with informed written consent and hospital ethics committee approval. The human stem cell study conformed to the 2013 WMA Declaration of Helsinki. As described in previously published articles from our laboratory (Kemp et al., 2010), bone marrow was taken at the time of total hip replacement surgery from the femoral shaft and placed into a sterile 50-ml tube containing 1,000 IU heparin. Patients with a history of malignancy, immune disorders, or rheumatoid arthritis were excluded from the study. Femoral shaft bone marrow donors were healthy, apart from osteoarthritis, and were not receiving drugs known to be associated with myelosuppression or bone marrow failure.

Femoral shaft marrow samples were broken up with a scalpel and washed with DMEM until the remaining material (bone) looked white at the bottom of the 50-ml tube. All washings were pipetted into a new 50-ml tube and kept for centrifugation. The suspension was centrifuged and resuspended in DMEM, overlaid onto an equal volume of Lymphoprep (density 1.077 ± 0.001 g/ml; Axis-Shield, Oslo, Norway), and centrifuged at 600*g* for 35 min at 24°C to separate the mononuclear cells from neutrophils and red cells. The mononuclear cell layer was harvested and washed twice in DMEM, and isolated MSCs were resuspended in MSC medium consisting of DMEM with 10% fetal bovine serum (StemCell Technologies, Vancouver, British Colombia, Canada) and 1% penicillin and streptomycin. Vented flasks (25 cm^2^) containing 10 ml MSC medium were seeded with 1 × 10^7^ cells for primary culture. Flasks were incubated at 37°C in a humidified atmosphere containing 5% CO_2_ and fed every week with MSC medium to remove nonadherent hematopoietic cells until the adherent fibroblast-like MSCs reached approximately 70% confluence. When they had reached confluence, cells were resuspended with 0.25% trypsin (Sigma-Aldrich) and reseeded at 2.25 × 10^5^ cells per flask (75 cm^2^) into first passage. Cultures were then incubated, fed every week with MSC medium, and, again, trypsinized; a cell count was taken and reseeded at 2.25 × 10^5^ cells per flask (75 cm^2^).

### MSC Characterization

Cells harvested from femoral shaft marrows displayed all the typical characteristics of MSCs in culture and were characterized as described in previously published articles from our laboratory (Wilkins et al., 2009; Kemp et al., 2010). Briefly, MSC cultures were characterized at third passage with anti-CD105, anti-CD45 (eBioscience, Hatfield, United Kingdom), anti-CD166, anti-CD90 (BD Biosciences, San Jose, CA), and anti-CD44 (Serotec, Kidington, United Kingdom). MSCs were also differentiated down the adipogenic, osteoblastic, and chondrogenic lineages.

### Preparation of MSC Transwell Cultures

Confluent MSC cultures, at third passage, were trypsinized and cultured in transwell inserts (Millipore, Billerica, MA) at 100,000 cells per well (24-well plate) or 400,000 cells per well (six-well plate) for 72 hr. Medium was then removed, MIN was added, and the transwells with MSCs were added to cortical neuron cultures 3 hr prior to the addition of DETANONOate.

### Statistical Analysis

Statistical analysis was carried out in GraphPad Prism (GraphPad Software, La Jolla, CA). Statistical comparisons were analyzed by one-tailed *t*-test or one-way ANOVA, with post hoc testing for comparisons between groups (Bonferroni test) when appropriate. Values are mean ± SE from at least three independent experiments. *P* < 0.05 represents statistical significance.

## RESULTS

### NO Reduces KIF5A and KIF21B Gene and Protein Expression in a Time-Dependent Manner

**Figure 1:**
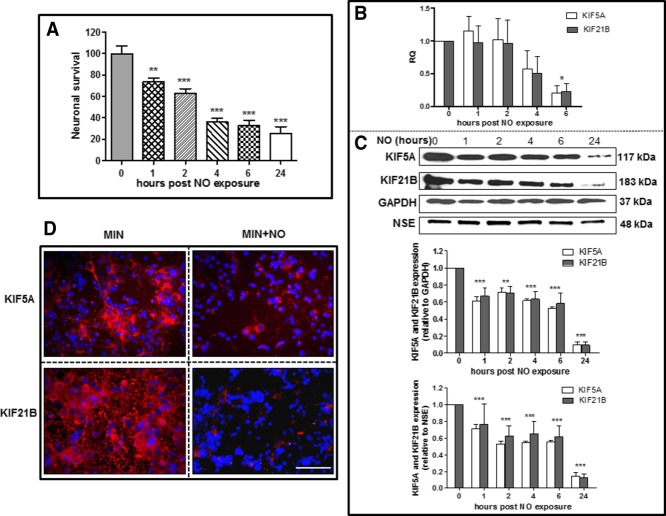
NO induces neuronal cell death (A) and decreases KIF5A/KIF21B gene (B) and protein (C) expression in rat cortical neurons in a time-dependent manner. 18s rRNA was used as a housekeeping gene for PCR, and KIF protein levels were normalized to GAPDH and NSE. Results are mean ± SE (n = 5 for RT-PCR experiments and n = 3 for Western blotting). *P < 0.05, **P < 0.01, ***P < 0.001. D: Immunocytochemistry for KIF proteins (red) and the nuclear marker DAPI (blue) was performed on neurons exposed to MIN or MIN with the addition of NO (MIN + N O) after 24 hr. Scale bar = 100 µm. [Color figure can be viewed in the online issue, which is available at wileyonlinelibrary.com.]

To investigate the effects of NO exposure on neuronal survival and KIF gene and protein expression, cortical neurons were maintained in B27-supplemented DMEM for 5 days before exposure to MIN with the NO donor DETANONOate for specific times. Cortical neurons were cultured in MIN alone (absence of DETANONOate), and values were expressed as a percentage of this baseline level. NO induced neuronal cell death in a time-dependent manner, with a significant reduction of neuronal survival from 1 hr of exposure to the maximum reduction after 24 hr of NO (*P* < 0.01 or *P* < 0.001; Fig. 1A).

After 1, 2, 4, and 6 hr of NO exposure, RNA was extracted to perform RT-PCR; 18s rRNA was used as a housekeeping gene. Analysis of KIF5A and KIF21B mRNA expression over a 6-hr period revealed that NO exposure resulted in a decrease of approximately 75% in both KIF5A and KIF21B mRNA expression in cortical neurons exposed to NO compared with exposure to MIN alone (*P* < 0.05; Fig. 1B). To determine the effect of NO exposure on KIF protein expression, cortical neurons were exposed to MIN with the addition of NO for 1, 2, 4, 6, and 24 hr, and after each time point proteins were extracted and Western blot analysis was performed. To determine the influence of neuronal death on KIF expression, densitometric analysis of the KIF Western blot bands was normalized to both total GAPDH and NSE levels. Results showed that NO caused a significant reduction in both KIF5A and KIF21B (*P* < 0.001) protein expression (relative to both GAPDH and NSE) in a time-dependent manner over a 24-hr period (Fig. 1C). After the first hour post-NO exposure, there was a significant decrease in protein expression of both KIF5A (approximately 40%) and KIF21B (approximately 30%), which, after 24 hr, reached a final reduction of approximately 80% for both proteins. A maximal KIF5A/KIF21B protein reduction was observed at 24 hr post-NO exposure, so subsequent NO exposure experiments with cortical neurons were performed with this time point. Immunocytochemistry for KIF5A, KIF21B, and the nuclear marker DAPI Vectashield was performed after 24 hr to confirm the Western blot results (Fig. 1D). KIF5A expression was localized in both the cell body and the axon of cortical neurons, but after NO exposure its expression was widely decreased, with nearly a complete absence; a small number of cell bodies still showed low KIF5A expression post-NO exposure. KIF21B expression was detected both in the cell body and in what appeared to be a network of dendrites. Additionally, NO exposure to neuronal cultures resulted in an extensive loss of KIF21B protein.

### NO Causes Reduction in Both Axonal Length and Neuronal Survival

**Figure 2:**
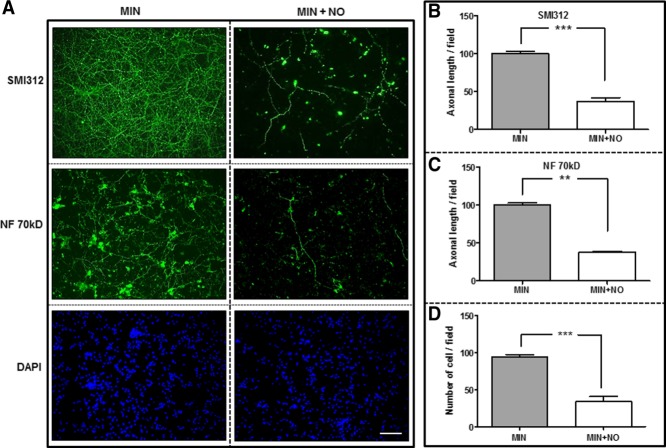
NO causes reductions in axon length and neuronal viability. Rat cortical neurons were exposed to MIN and MIN with the addition of NO for 24 hr. A: Cultures were stained for the nuclear marker DAPI (blue) and the panaxonal NF marker SMI312 (green) or NF 70 kD (green). Axonal length was measured by SMI312 (B) and NF 70 kD (C) labeling per culture field, and viable neurons (D) were counted by using DAPI positivity per field. Results are mean ± SE (n = 4). **P < 0.01, ***P < 0.001. Scale bar = 100 µm. [Color figure can be viewed in the online issue, which is available at wileyonlinelibrary.com.]

Cortical neurons were maintained in MIN alone or with the addition of NO donor for 24 hr. Cells were then fixed and stained for the panaxonal NF marker SMI312, the low-molecular-weight subunit of NF (NF 70 kD), βIII-tubulin (not shown), and the nuclear marker DAPI Vectashield (Fig. 2A). Axonal length was measured by SMI312 and NF 70 kD labeling, and viable neurons were counted by using DAPI/βIII-tubulin positivity per field. Cells with fragmented or abnormally shaped nuclei were excluded. Cortical neurons cultured with NO showed a significant reduction in phosphorylated NF length (*P* < 0.001; Fig. 2B), nonphosphorylated NF length (*P* < 0.01; Fig. 2C), and total live cells (*P* < 0.001; Fig. 2D) compared with control.

### NO Causes a Time-Dependent Loss of NF Phosphorylation

**Figure 3:**
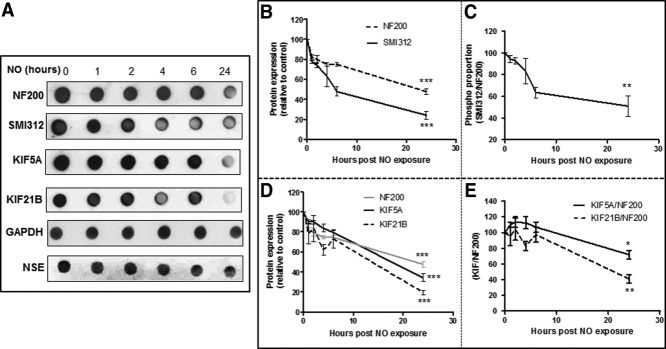
NO causes a time-dependent loss of NF phosphorylation and KIF expression. A: Representative immunodot blots for NF200, SMI312, KIF5A, KIF21B, GAPDH, and NSE in cortical neurons exposed to NO for different lengths of time. NO causes a time-dependent decrease in SMI312 and NF200 expression (B). Axonal phosphorylation (C) calculated by the ratio phosphorylated NF/total NF (SMI312/NF200) and KIF expression (D). E: KIF5A/NF200 and KIF21B/NF200 protein ratios in rat cortical neurons post-NO exposure. Both GAPDH and NSE were used as protein loading controls. Results are mean ± SE (n = 3). *P < 0.05, **P < 0.01, ***P < 0.001.

To analyze the effect of NO exposure on NF phosphorylation, dot blot protein expression analyses of phosphorylated NF (SMI312) and total NF (NF200) were performed (Fig. 3A). Cortical neurons were maintained in B27-supplemented DMEM for 5 days before exposure to NO donor for 1, 2, 4, 6, and 24 hr, and at each time point proteins were extracted and dot blot was performed. Cortical neurons were cultured in MIN alone (absence of NO), and values were compared with this baseline level. Equal loading was confirmed by GAPDH and NSE expression.

NO caused a time-dependent decrease in SMI312 and NF200 expression over a 24-hr period (*P* < 0.001; Fig. 3B), but the reduction of SMI312 was greater and occurred earlier than that of NF200. To calculate an index of total phosphorylated NF in axons after NO exposure, the ratio SMI312/NF200 (phosphorylated NF/total NF) was determined. With this analysis, it was demonstrated that NO caused a significant time-dependent decrease (*P* < 0.01) in NF phosphorylation over a 24-hr period (Fig. 3C).

### KIF Reduction Precedes the Loss of NF

To determine whether NO-induced reduction in KIF protein levels correlates with reduction in axonal structural protein levels, dot blots for the axon marker NF200 and KIF proteins were perform in cortical neurons after 1, 2, 4, 6, and 24 hr of NO exposure (Fig. 3A). Equal loading was confirmed by both GAPDH and NSE analysis. NSE was used in addition to GAPDH to ensure that changes in KIF levels were not solely a result of neuronal loss.

NO caused a time-dependent decrease of NF200, KIF5A, and KIF21B expression over a 24-hr period (*P* < 0.001; Fig. 3D). The ratios KIF5A/NF200 and KIF21B/NF200 were calculated at each time point, and results showed that both ratios decreased in a time-dependent manner, suggesting that KIF5A (*P* < 0.05) and KIF21B (*P* < 0.01) reduction precedes the loss of total NF200 (Fig. 3E).

### MSCs Preserve Axonal Length and Increase Survival in Cortical Neurons Exposed to NO

**Figure 4:**
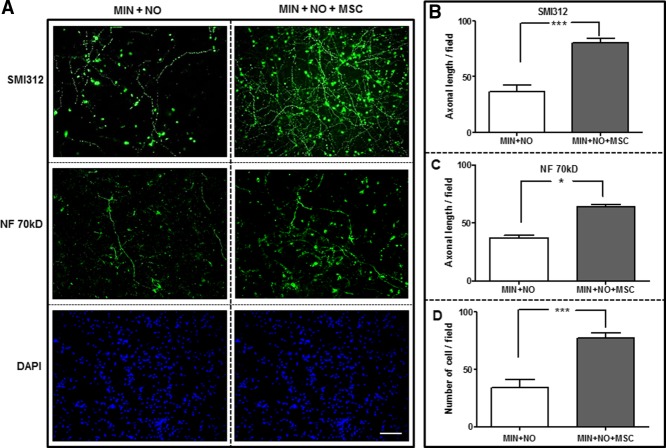
MSCs preserve axonal length and increase survival in rat cortical neurons exposed to NO. Cortical neurons were exposed to MIN with NO alone (MIN + NO) or with the addition of MSCs in a transwell coculture system (MIN + NO + MSC) for 24 hr. A: Cultures were stained for the nuclear marker DAPI (blue) and the panaxonal NF marker SMI312 (green) or NF 70 kD (green). Axonal length was measured by SMI312 (B) and NF 70 kD (C) labeling per culture field, and viable neurons (D) were counted by using the nuclear marker DAPI. Results are mean ± SE (n = 4). *P < 0.05, ***P < 0.001. Scale bar = 100 µm. [Color figure can be viewed in the online issue, which is available at wileyonlinelibrary.com.]

Cortical neurons were maintained in B27-supplemented DMEM for 5 days before exposure to MIN and NO with/without the addition of MSCs in a transwell coculture system, which allows the exchange of soluble factors without any direct contact between the neurons and MSCs. After 24 hr, neurons were fixed and stained for the panaxonal NF marker SMI312, NF 70 kD, βIII-tubulin (not shown), and the nuclear marker DAPI Vectashield (Fig. 4A). Axonal length was measured by SMI312 and NF 70 kD labeling, and viable neurons were counted by using DAPI/βIII-tubulin positivity per field. Both axonal length (Fig. 4B,C) and total live cells (Fig. 4D) were significantly increased in cocultures of MSC and cortical neurons exposed to NO compared with cortical neurons exposed to NO alone (*P* < 0.001, *P* < 0.05, respectively).

### Indirect Coculture of MSCs and Rat Cortical Neurons Preserves KIF5A and KIF21B Protein Expression From NO Exposure Without Increasing KIF Gene Expression

**Figure 5:**
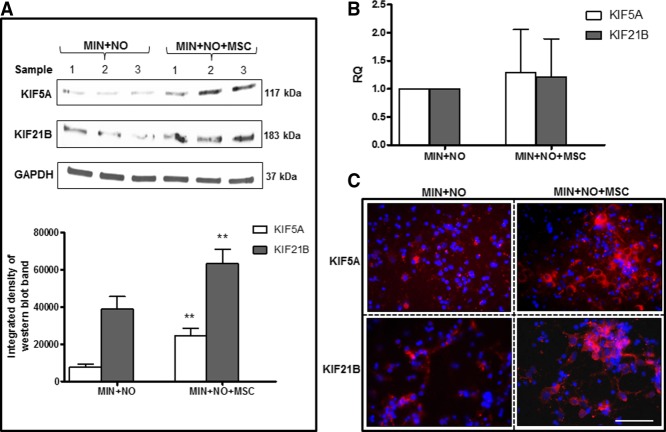
Indirect coculture of MSCs and rat cortical neurons preserves KIF5A and KIF21B protein expression from NO exposure. KIF5A and KIF21B protein expression with corresponding densitometric analysis (A) and mRNA expression (B) of cortical neurons exposed to NO alone (MIN + NO) or with the addition of MSCs in a transwell coculture system (MIN + NO + MSC). 18s rRNA was used as a housekeeping gene, and GAPDH level was used to control equal protein loading. Results are mean ± SE (n = 3 for Western blotting and n = 5 for RT-PCR experiments). **P < 0.01. C: Cortical neurons were exposed to NO with or without the addition of MSCs in a transwell coculture system for 24 hr, and cells were stained for KIF5A (red), KIF21B (red), and the nuclear marker DAPI (blue). Scale bar = 100 µm. [Color figure can be viewed in the online issue, which is available at wileyonlinelibrary.com.]

To investigate whether coculture of MSCs with cortical neurons was able to preserve KIF5A and KIF21B expression in neurons exposed to NO, cortical neurons were exposed to NO in the presence of MSCs seeded in a transwell system. KIF protein expression was analyzed 24 hr (Fig. 5A) post-NO exposure, and gene expression was analyzed 6 hr (Fig. 5B) post-NO exposure because previous results have shown a significant reduction in KIF protein expression after 24 hr and a significant reduction in gene expression after 6 hr. After 24 hr of NO exposure, cells were also fixed and stained for KIF5A, KIF21B, and the nuclear marker DAPI Vectashield (Fig. 5C).

For both KIF5A and KIF21B, protein expression (calculated by densitometric analysis of the Western blot bands) was significantly increased in cortical neurons exposed to NO and MSCs compared with NO alone (*P* < 0.01; Fig. 5A). No significant changes in the gene expression of KIF5A or KIF21B were observed among neurons exposed to NO alone or with the addition of MSCs in transwells (Fig. 5B). Immunofluorescence analysis confirmed that MSCs protect both KIF5A and KIF21B cortical neuronal expression from NO damage. KIF expression was seemingly lost when neurons were exposed to NO, whereas KIF labeling was preserved in several neuronal cells when exposed to NO in the presence of MSCs (Fig. 5C).

### MSCs Do Not Regulate KIF5A and KIF21B Protein Expression in the Absence of Toxic Insult

**Figure 6:**
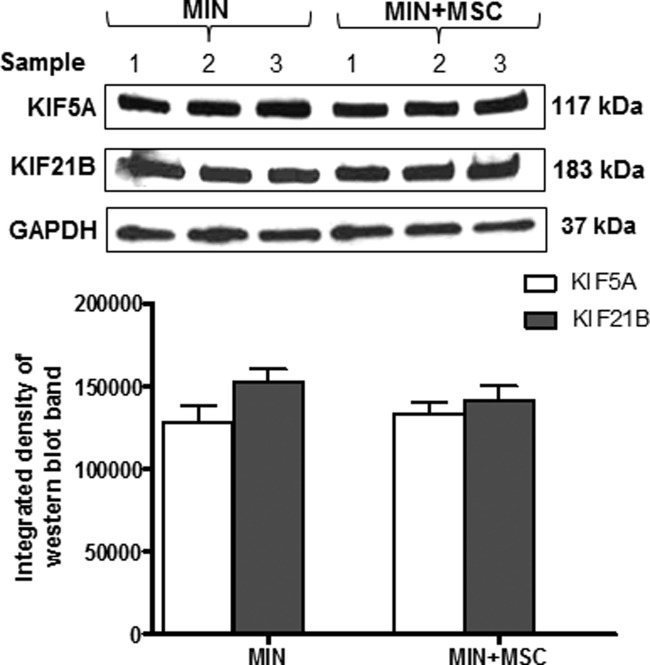
MSCs do not regulate KIF protein expression under standard culture conditions. KIF5A and KIF21B Western blot with corresponding densitometric analysis of rat cortical neurons exposed to MIN or indirect cocultures with MSCs (MIN + MSC) in the absence of toxic insult. Results are mean ± SE (n = 3).

To clarify whether MSCs regulate KIF levels in the absence of toxic insults, cortical neurons were maintained in MIN or cultured with MSC in a transwell coculture system (MIN + MSC), all without the addition of NO. After 24 hr, proteins were extracted, and Western blot analysis for KIF5A and KIF21B expression was performed. Densitometric analysis of the Western blot bands revealed no changes in KIF expression among the different conditions tested, suggesting that MSCs did not influence KIF levels under standard culture conditions (Fig. 6).

## DISCUSSION

Several studies have suggested a relationship between reactive oxygen and nitrogen species, abnormal protein accumulation, mitochondrial dysfunction, and neuronal cell injury; however, how nitrosative/oxidative stress causes axonal and neuronal injury is still not entirely clear. Axonal transport is vital for maintaining axonal integrity and neuronal survival, yet there is limited knowledge of how nitrosative/oxidative stress affects motor proteins and whether the reduction in axonal transport induced by nitrosative/oxidative stress is an early event that contributes to the progression of axonal injury. This study has investigated the effect of NO exposure on expression of kinesin superfamily members in rat cortical neurons and the relationship between KIF expression and axonal integrity and demonstrates that NO causes a time-dependent decrease in gene and protein expression of KIF5A and KIF21B in neurons and an overall reduction in axonal components. In addition, this study demonstrates that MSCs secrete soluble factors that protect neurons and axonal transport proteins.

NO is involved in several important functions in the central nervous system (CNS), including modulation of synaptic neurotransmission and plasticity, regulation of cerebral blood flow, and immune cell responses to pathogens (Bennaroch, 2011). However, disturbances in the normal redox state of cells and an overproduction of reactive nitrogen species play key roles in mediating inflammatory neuronal injury in several neurodegenerative diseases, including MS.

In this study, rat cortical neurons cultured with NO showed a significant reduction in axonal length (demonstrated by antibody labeling against phosphorylated and nonphosphorylated NF epitopes) and total live cells compared with controls. Several previous studies have demonstrated that NO is a key mediator of neuronal cell death through a variety of mechanisms, including energy depletion-induced necrosis by inhibition of mitochondrial respiration; glutamate release and subsequent excitotoxicity; p38 MAP kinase activation followed by Bax (a proapoptotic member of the Bcl-2 family) translocation and clustering; release of proapoptotic factors, including cytochrome c; and activation of p53 (Brown and Bal-Price, 2003; Yuan et al., 2007). Less clear, however, is how NO mediates axonal NF dephosphorylation. NFs are involved in defining structural and functional integrity of myelinated axons (Perrot et al., 2008) and are the most extensively phosphorylated proteins in neurons, with an intense phosphorylation in axons and little or no phosphorylation in cell bodies and dendrites (Nixon and Shea, 1992). Perturbations of NF metabolism and aberrant NF phosphorylation are associated with axon damage and are frequently observed in neurodegenerative diseases. The current results demonstrate that NO causes a time-dependent decrease in NF phosphorylation within axons. High levels of NF phosphorylation protect axons against proteolysis (Pant, 1988); therefore, dephosphorylation might render the axon more susceptible to degradation. Phosphorylation also controls axon caliber and interfilament spacing (Perrot et al., 2008), so dephosphorylation might decrease NF stability and render the axon more vulnerable to nitrosative stress.

In addition, abnormal aggregations of NF have been observed in several neurodegenerative disorders, implying disordered axonal transport. Anterograde transport of NF is mediated via kinesin molecular motors (Yabe et al., 2000), and disruption of KIF5A induces accumulation of NF in the cell bodies of peripheral sensory neurons (Xia et al., 2003). The current results demonstrate that high concentrations of NO cause reductions in KIF mRNA and protein expression that may contribute to axonal dysfunction and loss. In animal models of MS, Alzheimer's disease, and Parkinson's disease, KIF5 levels are significantly downregulated and are associated with NF, APP, and α-synuclein aggregations (Stokin et al., 2005; Chu et al., 2012; Kreutzer et al., 2012). Abnormalities of axonal transport seem to be a common pathological feature of several neurodegenerative diseases in which protein accumulation and spheroid formation contribute to the damaging effects, but the mechanisms causing these blockages are still unknown. Oxidative/nitrosative stress has also been implicated in many neurodegenerative disorders; therefore, our finding that NO decreases motor protein expression may be an important factor in nitrosative stress-induced axonal injury. Both heavy and light chains of KIFs are phosphorylated, and their phosphorylation state regulates their function (Sato-Yoshitake et al., 1992; Morfini et al., 2002). It is known that NO regulates a number of intracellular signaling pathways; therefore, under nitrosative stress, abnormal activation of protein kinases and alterations in protein phosphorylation could impair the functions of KIFs in several ways, by 1) preventing the binding of KIFs to microtubules, 2) preventing the binding between KIFs and their cargoes, 3) abolishing ATPase activity linked to KIFs that generates motile force, and 4) increasing susceptibility of KIFs to degradation by the proteasome system.

This study has determined that the ratio KIF/NF200 post-NO exposure decreases, suggesting that KIF reduction precedes the loss of NF. Impaired axonal transport might be an early event that can contribute, per se, to progression of injury because impairment of anterograde transport results in lack of the distribution of mitochondria, synaptic vesicles, NF, and APP, all critical for neuronal function.

Human bone marrow MSCs represent promising candidates for cell therapy because of their capacity to modulate immune responses and to produce trophic support factors (Uccelli et al., 2011). This study has analyzed the capacity of MSCs to promote neuronal cell survival in vitro in cortical neurons exposed to NO with the addition of MSCs in a transwell coculture system that allowed the exchange of soluble factors without any direct contact between neurons and MSCs. Both axonal length and total live neuron numbers were significantly increased in cocultures of MSC and rat cortical neurons exposed to NO compared with cortical neurons exposed to NO alone. Human MSCs have been shown to secrete neurotrophic factors, such as brain-derived neurotrophic factor (BDNF), nerve growth factor (NGF), glial cell line-derived neurotrophic factor (GDNF), and several neurite-inducing factors as well as axon guidance and neural cell adhesion molecules (Zhang et al., 2005; Crigler et al., 2006). BDNF has been shown to increase survival of neurons post-NO exposure through the activation of several cell survival pathways (Wilkins et al., 2009), NGF stimulates axonal repair in both acute and chronic CNS injury (Walsh et al., 1999), and GDNF has been shown to protect catecholaminergic and serotonergic neuronal perikarya from oxidative stress (Whone et al., 2012).

This study also demonstrates that coculturing MSCs in transwells leads to preservation of KIF5A and KIF21B protein expression in neurons exposed to NO. No significant changes in the gene expression of KIF5A and KIF21B were observed in NO-exposed neurons cocultured with MSCs in transwells, suggesting that factors released by MSCs are protecting KIFs rather than inducing their increased expression. This supports the current theory that the major protective effect of MSCs occurs through their capacity to secrete a range of potentially neuroprotective and antioxidant factors (Kemp et al., 2010). Furthermore, no difference in KIF expression was observed in cortical neurons cultured with MSCs in transwells without the addition of NO, suggesting that MSCs did not regulate KIF expression in the absence of toxic insult.

Altogether, our results provide a better understanding of potential mechanisms involved in the disruption of axonal transport and abnormal accumulation of proteins in axons during nitrosative insult. Intact axonal transport is required to maintain the overall metabolic balance, and function of the neuron and impaired axonal transport might not be only a consequence of axon damage but also a major contributory factor that leads to axonopathy. Moreover, the ability of MSCs to protect KIF proteins from NO damage provides further evidence of their therapeutic potential for neurological disorders of the CNS involving nitrosative injury as a result of their multiplicity of neuroprotective effects.
